# Supplementary material for: Unveiling the Bioactive Potential of the Invasive Jellyfish Phyllorhiza punctata Through Integrative Transcriptomic and Proteomic Analyses
Source: Biomolecules. 2025 Aug 4;15(8):1121. doi: 10.3390/biom15081121 (PMC12383608; doi:10.3390/biom15081121)
Supplement: Supplementary file 1 [file biomolecules-15-01121-s001.zip › Suplementary Material/FileS3/PEPFOLD4/XP_065059524_7680/clusters.html]

 PEP-FOLD clusters


Download archive of all models  here   

|  |  |  |
| --- | --- | --- |
| # | file | sOPEP |
| # cluster 1 - Size: 24 |  |  |
|  | XP\_065059524\_7680-model1 | -21.469 |
|  | XP\_065059524\_7680-model1.1 | -21.2419 |
|  | XP\_065059524\_7680-model1.2 | -21.2284 |
|  | XP\_065059524\_7680-model1.3 | -21.1865 |
|  | XP\_065059524\_7680-model1.4 | -21.1326 |
|  | XP\_065059524\_7680-model1.5 | -21.1248 |
|  | XP\_065059524\_7680-model1.6 | -21.1197 |
|  | XP\_065059524\_7680-model1.7 | -21.1164 |
|  | XP\_065059524\_7680-model1.8 | -21.089 |
|  | XP\_065059524\_7680-model1.9 | -21.0836 |
|  | XP\_065059524\_7680-model1.10 | -21.0619 |
|  | XP\_065059524\_7680-model1.11 | -21.0619 |
|  | XP\_065059524\_7680-model1.12 | -21.0405 |
|  | XP\_065059524\_7680-model1.13 | -21.0254 |
|  | XP\_065059524\_7680-model1.14 | -21.012 |
|  | XP\_065059524\_7680-model1.15 | -20.9784 |
|  | XP\_065059524\_7680-model1.16 | -20.9631 |
|  | XP\_065059524\_7680-model1.17 | -20.9422 |
|  | XP\_065059524\_7680-model1.18 | -20.9408 |
|  | XP\_065059524\_7680-model1.19 | -20.9266 |
|  | XP\_065059524\_7680-model1.20 | -20.9197 |
|  | XP\_065059524\_7680-model1.21 | -20.9078 |
|  | XP\_065059524\_7680-model1.22 | -20.9055 |
|  | XP\_065059524\_7680-model1.23 | -20.8885 |
| # cluster 2 - Size: 17 |  |  |
|  | XP\_065059524\_7680-model2 | -21.3388 |
|  | XP\_065059524\_7680-model2.1 | -21.2382 |
|  | XP\_065059524\_7680-model2.2 | -21.227 |
|  | XP\_065059524\_7680-model2.3 | -21.155 |
|  | XP\_065059524\_7680-model2.4 | -21.153 |
|  | XP\_065059524\_7680-model2.5 | -21.1495 |
|  | XP\_065059524\_7680-model2.6 | -21.1368 |
|  | XP\_065059524\_7680-model2.7 | -21.1195 |
|  | XP\_065059524\_7680-model2.8 | -21.1141 |
|  | XP\_065059524\_7680-model2.9 | -21.1039 |
|  | XP\_065059524\_7680-model2.10 | -21.0955 |
|  | XP\_065059524\_7680-model2.11 | -21.066 |
|  | XP\_065059524\_7680-model2.12 | -21.04 |
|  | XP\_065059524\_7680-model2.13 | -21.0278 |
|  | XP\_065059524\_7680-model2.14 | -20.9613 |
|  | XP\_065059524\_7680-model2.15 | -20.9192 |
|  | XP\_065059524\_7680-model2.16 | -20.8679 |
| # cluster 3 - Size: 33 |  |  |
|  | XP\_065059524\_7680-model3 | -21.3366 |
|  | XP\_065059524\_7680-model3.1 | -21.3195 |
|  | XP\_065059524\_7680-model3.2 | -21.3181 |
|  | XP\_065059524\_7680-model3.3 | -21.257 |
|  | XP\_065059524\_7680-model3.4 | -21.2426 |
|  | XP\_065059524\_7680-model3.5 | -21.2281 |
|  | XP\_065059524\_7680-model3.6 | -21.2281 |
|  | XP\_065059524\_7680-model3.7 | -21.2211 |
|  | XP\_065059524\_7680-model3.8 | -21.1903 |
|  | XP\_065059524\_7680-model3.9 | -21.167 |
|  | XP\_065059524\_7680-model3.10 | -21.1434 |
|  | XP\_065059524\_7680-model3.11 | -21.1401 |
|  | XP\_065059524\_7680-model3.12 | -21.1023 |
|  | XP\_065059524\_7680-model3.13 | -21.0874 |
|  | XP\_065059524\_7680-model3.14 | -21.0691 |
|  | XP\_065059524\_7680-model3.15 | -21.0634 |
|  | XP\_065059524\_7680-model3.16 | -21.0578 |
|  | XP\_065059524\_7680-model3.17 | -21.0532 |
|  | XP\_065059524\_7680-model3.18 | -20.9928 |
|  | XP\_065059524\_7680-model3.19 | -20.9784 |
|  | XP\_065059524\_7680-model3.20 | -20.9671 |
|  | XP\_065059524\_7680-model3.21 | -20.9436 |
|  | XP\_065059524\_7680-model3.22 | -20.9367 |
|  | XP\_065059524\_7680-model3.23 | -20.933 |
|  | XP\_065059524\_7680-model3.24 | -20.9051 |
|  | XP\_065059524\_7680-model3.25 | -20.8927 |
|  | XP\_065059524\_7680-model3.26 | -20.873 |
|  | XP\_065059524\_7680-model3.27 | -20.8695 |
|  | XP\_065059524\_7680-model3.28 | -20.8644 |
|  | XP\_065059524\_7680-model3.29 | -20.8311 |
|  | XP\_065059524\_7680-model3.30 | -20.8041 |
|  | XP\_065059524\_7680-model3.31 | -20.7888 |
|  | XP\_065059524\_7680-model3.32 | -20.6712 |
| # cluster 4 - Size: 11 |  |  |
|  | XP\_065059524\_7680-model4 | -21.3179 |
|  | XP\_065059524\_7680-model4.1 | -21.1935 |
|  | XP\_065059524\_7680-model4.2 | -21.1775 |
|  | XP\_065059524\_7680-model4.3 | -21.128 |
|  | XP\_065059524\_7680-model4.4 | -21.1241 |
|  | XP\_065059524\_7680-model4.5 | -21.0567 |
|  | XP\_065059524\_7680-model4.6 | -21.0211 |
|  | XP\_065059524\_7680-model4.7 | -21.0105 |
|  | XP\_065059524\_7680-model4.8 | -20.9865 |
|  | XP\_065059524\_7680-model4.9 | -20.9734 |
|  | XP\_065059524\_7680-model4.10 | -20.8395 |
| # cluster 5 - Size: 4 |  |  |
|  | XP\_065059524\_7680-model5 | -21.3114 |
|  | XP\_065059524\_7680-model5.1 | -20.9753 |
|  | XP\_065059524\_7680-model5.2 | -20.9374 |
|  | XP\_065059524\_7680-model5.3 | -20.9166 |
| # cluster 6 - Size: 11 |  |  |
|  | XP\_065059524\_7680-model6 | -21.2186 |
|  | XP\_065059524\_7680-model6.1 | -21.1006 |
|  | XP\_065059524\_7680-model6.2 | -21.0605 |
|  | XP\_065059524\_7680-model6.3 | -21.0593 |
|  | XP\_065059524\_7680-model6.4 | -21.0555 |
|  | XP\_065059524\_7680-model6.5 | -20.9599 |
|  | XP\_065059524\_7680-model6.6 | -20.9231 |
|  | XP\_065059524\_7680-model6.7 | -20.9154 |
|  | XP\_065059524\_7680-model6.8 | -20.8642 |
|  | XP\_065059524\_7680-model6.9 | -20.8507 |
|  | XP\_065059524\_7680-model6.10 | -20.7694 |
|  |  |  |
